# Unveiling the future: precision pharmacovigilance in the era of personalized medicine

**DOI:** 10.1007/s11096-024-01709-x

**Published:** 2024-02-28

**Authors:** Lurdes Silva, Teresa Pacheco, Emília Araújo, Rita J. Duarte, Inês Ribeiro-Vaz, Renato Ferreira-da-Silva

**Affiliations:** 1 Faculty of Pharmacy of the University of Porto, Porto, Portugal; 2 Palliative Care Service, Portuguese Oncology Institute of Porto (IPO Porto), Porto, Portugal; 3 Center for Health Technology and Services Research, Associate Laboratory RISE – Health Research Network (CINTESIS@RISE), Porto, Portugal; 4 Pharmacovigilance, Bayer Portugal, Lisbon, Portugal; 5 Porto Pharmacovigilance Centre, Faculty of Medicine of the University of Porto, Porto, Portugal; 6 Department of Community Medicine, Health Information and Decision, Faculty of Medicine of the University of Porto, Porto, Portugal

**Keywords:** Drug safety, Patient outcomes, Personalized medicine, Pharmacogenomics, Pharmacovigilance, Precision medicine

## Abstract

In the era of personalized medicine, pharmacovigilance faces new challenges and opportunities, demanding a shift from traditional approaches. This article delves into the evolving landscape of drug safety monitoring in the context of personalized treatments. We aim to provide a succinct reflection on the intersection of tailored therapeutic strategies and vigilant pharmacovigilance practices. We discuss the integration of pharmacogenetics in enhancing drug safety, illustrating how genetic profiling aids in predicting drug responses and adverse reactions. Emphasizing the importance of phase IV—post-marketing surveillance, we explore the limitations of pre-marketing trials and the necessity for a comprehensive approach to drug safety. The article discusses the pivotal role of pharmacogenetics in pre-exposure risk management and the redefinition of pharmacoepidemiological methods for post-exposure surveillance. We highlight the significance of integrating patient-specific genetic profiles in creating personalized medication leaflets and the use of advanced computational methods in data analysis. Additionally, we examine the ethical, privacy, and data security challenges inherent in precision medicine, emphasizing their implications for patient consent and data management.

## Introduction

The assurance of medicines' safety stands as a cornerstone within the spectrum of healthcare. It is a vigilant and ongoing process that traverses every phase of a drug's existence, from its inception to its real-world application [1]. Pharmacovigilance, the science and activities relating to the detection, assessment, understanding, and prevention of adverse effects or any other medicine-related problem, plays a critical role in this process. The journey of drug development involves meticulous safety assessments. In the preliminary stages, as drugs undergo pre-clinical studies, the focus is on establishing a secure dosage for human use and defining safety parameters crucial for clinical oversight. In the intricate journey of drug development, the pivotal phase IV—post-marketing surveillance—takes the centre stage as the keystone in assessing the safety of medicines [2, 3]. Serving as a bridge between pre-market clinical trials and real-world application, phase IV offers a comprehensive lens to evaluate a drug's long-term safety profile and unveil rare adverse drug reactions (ADR) [4, 5].

While pre-marketing trials provide insights, they have limitations that post-marketing surveillance addresses. Controlled trials may not fully reveal a drug's impact on diverse patients and might miss long-term adverse reactions. This highlights phase IV's importance, examining drug safety in real-world complexity [5]. This transition propels us into an examination of the evolving pharmacovigilance landscape within the precision medicine (PM) era [6]. Within this article, we will succinctly reflect on the challenges and opportunities that arise, uniquely focusing on the intersection of tailored treatments and vigilant drug safety.

## Precision medicine: pioneering a paradigm shift

Traditionally, medical interventions followed a one-size-fits-all approach, applying the same treatment to patients with identical conditions. However, the advent of PM challenges this paradigm [6, 7]. PM, also referred to as “personalized medicine”, represents a cutting-edge approach to customizing disease prevention and treatment strategies. It considers the unique genetic makeup, environmental factors, and lifestyle choices of individuals. The primary aim of this approach is to accurately align treatments with the specific needs of patients, ensuring that the correct therapies are administered to the appropriate individuals at the most opportune moments [8]. This approach aims to personalize healthcare, where medical decisions, treatments, practices, or products are individually adapted to each patient, moving away from the traditional one-size-fits-all approach in medicine. This methodology finds profound implications within the realm of epilepsies, where PM's substantial achievements have been widely documented, occasionally showcasing remarkable results. Notably, significant strides in epilepsy genetics have propelled advancements in this field [9, 10]. The cornerstone of PM lies in personalization, elevating patients to the forefront of treatment decisions. This approach emphasizes the importance of genetics in understanding individual disease risks and medication responses, central to precision medicine's goal of tailoring treatments for optimal efficacy and minimal adverse reactions [8, 11].

As the influence of PM extends notably into the fields of oncology, rare diseases, chronic conditions, and infectious diseases, its transformative implications become increasingly apparent. For instance, the introduction of antiretroviral therapy (ART) has revolutionized human immunodeficiency virus (HIV) management, shifting it from a once-debilitating condition to a manageable chronic illness [12]. This paradigm shifts prompts reflections on long-term quality of life for ART recipients and the safety of these drugs, such as efavirenz, a widely used medication that has been associated with neuropsychiatric ADR [12]. Similarly, strides in understanding type 2 diabetes encompass human genetics, biomarkers, and clinical trials, fostering the integration of this knowledge into clinical practice. Emerging technologies like single-cell RNA have unravelled cellular complexities in diseases like atherosclerosis, offering insights that could refine therapeutic strategies [13]. Moreover, the ascendancy of immunotherapy in cancer treatment accentuates the importance of identifying biomarkers to predict immune-related ADR [14]. For example, in patients with non-small-cell lung cancer treated with PD-1/PD-L1 inhibitors, certain genetic markers have been identified as potential predictors for the development of checkpoint inhibitor pneumonitis, allowing for preemptive monitoring and management.

## Redefining therapeutic safety surveillance in the era of precision medicine

While several examples illustrate the dynamic nature of PM and its potential to shape innovative treatments, this also signals a fundamental shift in the safety monitoring paradigm. As each patient receives a unique, targeted, and personalized treatment specific to their clinical condition, there arises a pressing need to rethink traditional pharmacovigilance methods. The current approaches, many rooted in classic pharmacoepidemiological study designs, must be adapted to meet the demands of monitoring treatment safety in an era of highly individualized medicine (Table 1). As such, we are witnessing the emergence of a concept known as “precision pharmacovigilance”, which merges drug safety monitoring with the tailored approach of PM. This concept aims to ensure that drug safety surveillance is closely aligned with the individual health needs of each patient. By adopting advanced data collection and analysis methods, precision pharmacovigilance has the potential to transform care delivery sites into crucial hubs for aggregating and utilizing patient-specific data for informed treatment decisions. Although still in its developmental phase and not yet widely recognized, this approach marks a significant stride in prioritizing the unique drug safety needs of individual patients. Furthermore, the ultimate objective of pharmacovigilance remains the same: to safeguard public health by preventing harm from ADR and ensuring the safe, effective, and rational use of medicines in diverse populations. This aligns with the overarching goal of enhancing patient outcomes and maintaining trust in healthcare systems and drug therapies. Considering the potential for integrating pharmacovigilance into PM, we propose two approaches in the surveillance of PM: (A) Prevention and Minimization of Pre-Exposure Risk, and (B) Post-Exposure Surveillance and Pharmacoepidemiology Research.

**Table 1:** Examples of recommendations and implementation strategies for advancing pharmacovigilance in precision medicine

Recommendations	Implementation strategies	Examples
Incorporate pharmacogenetics into routine clinical practice to optimise therapeutic outcomes	Integrate patient genetic data into pharmacological treatment management plans, including preventing adverse reactions, especially in polypharmacy and chronic disease patients, through pharmacogenetic or pharmacogenomic testing	The implementation of the GeneSight pharmacogenomic test in outpatient psychiatric care allowed for personalized medication management for major depression, where its application guided medication choices, resulting in significantly improved patient outcomes compared to unguided treatment decisions [23]
Implement large interoperable and longitudinal cohorts	Conduct cohort studies to analyse if the incidence of adverse reactions differs among subgroups of individuals with specific risk factors like genetic alterations compared to individuals without these alterations, thereby identifying risk factor responsibility for characteristics	The PreMed study in Finland, a register-based cohort analysis, linked genetic data from biobanks with national registries and healthcare data to study the pharmacogenetics of antithrombotic medications, such as warfarin, by monitoring drug reactions based on VKORC1 and CYP2C9 gene variants [24]
Invest in big data and artificial intelligence (AI) for advanced pharmacovigilance analytics	Develop and integrate classic pharmacoepidemiological methods with sophisticated data collection and analysis techniques using and big data, enhancing real-time monitoring and decision-making in pharmacovigilance	In South Korea, the application of data mining methods to the Korea Adverse Event Reporting System (KAERS) database exemplifies the use of big data and AI in pharmacovigilance. This approach, aimed at detecting adverse events of doxycycline, utilized algorithms to analyze reporting ratios and compare detected signals against drug labels in multiple countries [25]
Utilize electronic health records as a source of phenotypic and genotypic information	Leverage the availability of these data for patient monitoring and adverse reaction prevention, combining them with additional patient-provided data	The EHR-Phenolyzer study, conducted by Columbia University and the Mayo Clinic, utilized electronic health records as a key source of phenotypic data. By integrating EHRs with whole-exome sequencing data, the study demonstrated enhanced efficiency in genetic diagnosis [26]
Focus on biomarker selection and validation	Implement robust methodologies to select and validate biomarkers, considering their prognostic and predictive values. A prognostic biomarker might indicate a patient's risk for drug-induced adverse reactions through gene expression changes, like specific microRNA detection. Predictive biomarkers, such as a somatic mutation in a specific gene, can signal a patient’s likelihood of ADR to certain drugs, guiding safer medication choices	The CorLipid trial, a post-hoc analysis involving patients with coronary artery disease and comorbid diabetes mellitus, highlights the potential of biomarker-based strategies in enhancing disease diagnosis and management. This study focused on metabolomic biomarkers to predict major cardiovascular events and assess coronary disease complexity [27]
Develop pharmacovigilance frameworks	Jointly develop guidance among various stakeholders (e.g., regulatory bodies, healthcare providers, pharmaceutical companies) for monitoring new therapeutics, particularly addressing limitations and biases typically associated with extrapolating classic pharmacoepidemiological methods (population-based) to individual-centric analysis in precision medicine	A proposal funded in response to a competitive proposal to the International Society for Pharmacoepidemiology, exemplifies a pioneering framework in pharmacovigilance development. It showcases how integrating pharmacoepidemiological methods with precision medicine can enhance drug safety and effectiveness, providing a strategic model for advancing personalized pharmacovigilance practices [6]

### Prevention and minimization of pre-exposure risk 

Currently, the approach that has been most discussed in the field of safety in PM is pharmacogenetics. Pharmacogenetics is a scientific discipline that explores how unique genetic variations in individuals significantly influence their responses to medications, as well as the potential for varying therapeutic effects and the occurrence of ADR, thereby guiding more personalized and effective medical treatments. Based on the premise that genetic markers can predict the safety of many drugs, pharmacogenetics will be crucial, as ADR could be preventable through recording personal pharmacogenetic profiles, providing individual recommendations for the use or avoidance of certain drugs, or customizing dosage regimens for patients with specific genotypes [15]. Building on this, the logical evolution of pharmacogenetics extends to the creation of personalized package leaflets, a revolutionary advance in personalized medicine. These inserts would be specifically tailored for each patient, integrating evidence generated from their pharmacogenetic profiles. This would enable the inclusion of detailed information about warnings, precautions, as well as potential ADR based on the individual genetics. Bayesian inference methods can be employed, aggregating all these data, generating new evidence, and aiding in clinical decision-making [16]. Despite the challenges in patient risk management due to potential errors or even malicious personalization, the personalized package insert serves as a precise guide for healthcare professionals and patients. It optimizes the safety and efficacy of treatment by tailoring pharmacological therapies to the unique genetic characteristics of each individual. This approach not only improves clinical management but also enhances patient awareness for potential risks associated with their specific medication therapies [17].

### Post-exposure surveillance and pharmacoepidemiology research

Although pharmacogenetics plays an important role in predicting safety issues on an individualized basis, it primarily focuses on a pre-exposure approach to treatment. The real question that arises is the redefinition of a new framework for pharmacoepidemiological methods specifically tailored for PM [6]. Pharmacoepidemiology is a scientific discipline that applies epidemiological methods to assess the use, benefits, and risks of medical products and interventions in human populations. Herein lies the first paradox. These methods, whether descriptive or analytical, were developed for application to populations sharing exposure to a particular medication, aiming to assess the development of ADR as an outcome (cohort studies) [17]. 

The concept of precision pharmacovigilance is poised to rely on innovative data collection and analysis methods [7]. Data collection should be based on electronic health records (EHR), drug exposure registries, internet of things devices for continuous patient monitoring, among other clinical data sources. Data analysis in PM should prioritize advanced computational and statistical methods, addressing biases in small, heterogeneous patient subgroups. It is also crucial to ensure sufficient statistical power, often requiring large consortia, especially in genetic studies where multiple testing corrections like the false discovery rate are vital. The timing and interpretation of genomic biomarker testing further add to this complexity, emphasizing the need for robust and nuanced analysis. This is due to each individual having a unique clinical condition and treatment regimen distinct from others. Bayesian inference, knowledge engineering, and various aspects of artificial intelligence (AI) are likely to be the most supportive in this context. These approaches can aggregate all these data, generating new evidence and aiding in clinical decision-making.

Employing machine learning and AI, pharmacovigilance can proactively observe patient reactions to medications, identify safety indicators, and dynamically adjust treatment methodologies. In line with this approach, yet equally vital, is the emphasis on passive surveillance. This method, encompassing case series as well, is predominantly characterised by the spontaneous reporting of ADR, thereby integrating the therapeutic recipient's experience, and placing the patient (and their caregivers) at the first line of new safety data collection. Active pharmacovigilance, as opposed to passive surveillance, aims to comprehensively ascertain the number of ADR through a continuous, pre-planned process. The methods outlined in the International Council for Harmonisation Topic E2E Pharmacovigilance Planning guidelines detail registries, sentinel sites, and drug event monitoring as the principal methods [18]. While active approaches are not limited to these three, these strategies, when suitably adapted to the new challenges of PM, can be effective methods for active surveillance focused on each patient. More than the chosen pharmacovigilance method, the monitoring approach and data sources will condition the success of the surveillance of these patients.

Regardless of the methods used, patient stratification will undoubtedly be a cornerstone in redefining these classic monitoring and data analysis methods. This stratification process, which involves segmenting a patient cohort into distinct subgroups where each represents a specific segment of the broader patient population, is accomplished by considering a range of factors. These factors include genetic background, physical attributes, coexisting conditions, omic data, polypharmacy, previous ADR, allergies, and other variables. In parallel, PM studies typically focus more intensively on using genomic information to define these populations and understand how this information impacts treatment effects. This approach ensures a more nuanced and comprehensive understanding of how treatments interact with individual patient characteristics [6]. This extensive data collection facilitates the division of patients into distinct groups, each characterized by unique epidemiological traits of their disease [7, 19]. One aspect of particular interest in these pharmacovigilance studies in PM is the selection of the biomarker to be studied and determining whether it is prognostic or predictive. The impact of these two types of markers can vary significantly [20]. For instance, associated genomic markers may not be consistently applicable across different populations or ethnic groups. This variability underscores the importance of careful biomarker selection and analysis to ensure accurate and relevant outcomes in PM research [21].

Furthermore, it is crucial to address the ethical and social challenges that pharmacovigilance faces, especially concerning access to genomic and other sensitive data, and the issues related to their dissemination in the context of patient monitoring. For example, in an instance of phenotype data attack, an unauthorized entity could inappropriately access an individual's genomic data and infer sensitive phenotypes such as disease susceptibilities. Even with certain genetic markers masked, there remains a risk of an attacker restoring the original genomic information through genotype imputation. This situation highlights the pressing need for stringent safeguards in pharmacovigilance practices to prevent such violations and protect the confidentiality and integrity of sensitive genetic data [22]. Patients may also face complex choices in PM and require assistance to comprehend the implications of their genetic data. They need help understanding the therapeutic choices made, as well as the outcomes and causality attributions associated with reported cases. The principles of autonomy, privacy, and informed consent also become paramount. Ensuring these principles involves not only providing patients with precise, clear information about how their data will be used but also genuinely understanding and respecting their decisions regarding the use of their personal health information. This is particularly critical when dealing with genomic data, which can reveal extensive personal health details. It is essential that patients are fully informed and have given explicit consent before their genomic data is utilized for pharmacovigilance purposes. Training pharmacoepidemiologists in the field of PM and fostering integrated collaboration among regulatory agencies, payers, healthcare systems, healthcare professionals, pharmaceutical companies, and patient representatives is essential to navigate these complexities. Lastly, while resource-limited settings face inherent challenges in accessing precision medicine and consequently in developing robust pharmacovigilance, leveraging available technologies and international collaboration offers a practical path to strengthen these essential healthcare systems.

In conclusion, pharmacovigilance in the era of PM is an ever evolving and dynamic field. As we continue to unlock the potential of genetic data, big data analytics, and patient-centered approaches, it is essential to remain vigilant about accompanying ethical and safety considerations while adopting new real-time surveillance, data collection, and analysis methods to finely tailor strategies to each drug's profile. This comprehensive approach not only advances patient care by adapting treatments to individual needs but also upholds the highest standards of safety in PM, reflecting the evolving nature of drug monitoring in this innovative era. However, to further enhance precision pharmacovigilance, there is a critical need for ongoing research and development, emphasizing collaborative efforts for future innovations in this field.
